# The heart of plastic: utilizing the *Drosophila* model to investigate the effects of micro/nanoplastics on heart function

**DOI:** 10.3389/ftox.2024.1438061

**Published:** 2024-08-16

**Authors:** Alyssa M. Hohman, Rachel M. Sorensen, Boris Jovanovic, Elizabeth M. McNeill

**Affiliations:** ^1^ Department of Food Science and Human Nutrition, Iowa State University, Ames, IA, United States; ^2^ Interdepartmental Program in Genetics and Genomics, Iowa State University, Ames, IA, United States; ^3^ Department of Ecology, Evolution, and Organismal Biology, Ames, IA, United States

**Keywords:** *Drosophila*, microplastic, nanoplastic, heart, cardiovascular system

## Abstract

Microplastics (MPs) and nanoplastics (NPs) have increasingly been found in the environment. Until recently, most MPs/NPs toxicological research has been done in aquatic systems resulting in a gap in knowledge regarding terrestrial systems. Plastics have been shown to enter the circulatory system of humans, and can accumulate within organs, little is known about the effect this has on health. Heart disease is the leading cause of death globally, so it’s critical to understand the possible impacts MPs/NPs have on the heart. The *Drosophila* model has been growing in popularity within the toxicology field, it allows for affordable and rapid research on the impacts of a variety of toxins, including plastics. Some research has examined toxicological effects of plastics on the fly, evaluating the effects on mortality, fecundity, development, and locomotion. However, no one has studied the effects on the *Drosophila* heart. We utilize the *Drosophila* model to identify the potential effects of oral exposure to polystyrene MPs (1 µm in diameter) and NPs (0.05 µm in diameter) particles on heart function. Flies were exposed to 1.4 × 10^11^ particles/d/kg of larvae for MPs and 1.2 × 10^18^ particles/d/kg of larvae for NPs from egg to pupal eclosion. Heart function was then analyzed utilizing semi-intact dissections and Semi-automatic Optic Heartbeat Analysis software (SOHA). Following exposure to MPs and NPs we see sexually dimorphic changes to heart size and function. This study highlights the importance of additional *Drosophila* MPs/NPs research to identify the molecular mechanisms behind these changes.

## 1 Introduction

### 1.1 Micro/nanoplastics

The presence of plastic in the environment is an emerging global concern. It’s estimated that between the 1950s-2015, approximately 8,300 million metric tons of plastic were manufactured, and of this, 4,900 million metric tons have been discarded and now reside in landfills or the natural environment (Geyer et al., 2017). The synthesis of plastic has continued to increase, approximately 36 million tons of plastic waste are generated each year in the United States alone (Dai et al., 2023). Plastics are long chains of polymers that are ideal for products due to their malleability (GESAMP, 2015) that can be created in various shapes and sizes. After long periods, plastic may degrade and fracture into smaller pieces. In this paper, MPs are any plastic greater than 100 nm but less than 5 mm in size, NPs are less than 100 nm in size.

Despite the pressing concern for the environment, the majority of MPs and NPs research has focused on the marine environment–especially fish (Blettler et al., 2018; Jacob et al., 2020; Romero-Becerra et al., 2020). There is great importance in evaluating the effects of MPs and NPs in terrestrial ecosystems, especially as the contamination for MPs in the European Union is conservatively estimated to be between 4–23 times higher for the terrestrial and freshwater environments than for marine environments (Horton et al., 2017). Emerging studies have evaluated the potential toxicity of MPs and NPs on terrestrial organisms such as earthworms, birds, mammals, insects, etc. (Huerta Lwanga et al., 2016; Buteler et al., 2022; de Souza et al., 2022; Sherlock et al., 2022). Yet this work is in its infancy and many questions remain about the specific impacts of this toxicity on an organism. Using mammals to study the toxicological impacts of MPs and NPs is expensive and raises ethical concerns, highlighting the need for a more accessible model organism.

### 1.2 Drosophotoxicology

*Drosophila melanogaster* are an affordable model with highly tractable genetics and a relatively short lifespan. This makes them an excellent model for a variety of research applications including toxicology research, so much so there is an entire field dedicated to drosophotoxicology (Chifiriuc et al., 2016). *Drosophila* have been utilized to evaluate the toxicity of metal nanoparticles which include gold, silver, titanium dioxide, carbon nanotubes (Ahamed et al., 2010; Pompa et al., 2011; de Andrade et al., 2014; Alaraby et al., 2016; Jovanović et al., 2016; 2018; Cvetković et al., 2020). In recent years the effects of NPs/MPs toxicity have been investigated using *Drosophila* (Adolfsson et al., 2018; Zhang et al., 2020; Demir, 2021; Jimenez-Guri et al., 2021; Matthews et al., 2021; Shen et al., 2021; Liang et al., 2022; Liu et al., 2022a; Zhong et al., 2022; Alaraby et al., 2023; Demira and Turna Demir, 2023; Kholy and Al Naggar, 2023; Tang et al., 2023). Two studies have evaluated whether MPs and NPs exposure alter lifespan in *Drosophila* using Polyethylene terephthalate (PET) and PS (Liang et al., 2022; Kholy and Al Naggar, 2023). Exposure to 2.5 µm PET (1,000, 10,000; and 20,000 ppm) using a log-rank test revealed no change in lifespan for female flies and surprisingly an increased lifespan for males exposed to 1,000 ppm (Liang et al., 2022). For 2 μm PS MPs, lifespan decreased at 0.005, 0.05, and 0.5 ppm (Kholy and Al Naggar, 2023).

### 1.3 Toxicology and the heart

Studies conducted in zebrafish larvae have demonstrated that NPs can enter circulation and accumulate in various organs during development, including the liver, pancreas, stomach and intestines, gallbladder, and the heart, where the highest concentration of particles was found in the pericardial sac (Pitt et al., 2018). This study also established that even at lower concentrations of NPs (0.1 ppm), where the plastics didn’t accumulate in the heart tissue, still showed cardiac dysfunction, which suggests that there are molecular mechanisms underlying the dysfunction.

Earlier this year, a study published in the New England Journal of Medicine revealed that in patients where MPs/NPs were detected in carotid artery plaques were at a significantly higher risk of heart attack, stroke, or death from any cause than those without (Marfella et al., 2024). This study also demonstrated that patients with additional health concerns, like diabetes, were at an even higher risk of negative outcomes due to MPs exposure. Finally, they highlighted the need for standardized *in vivo* models, utilizing environmentally derived plastics, to better understand the mechanism behind the cardiovascular phenotypes seen because of MPs/NPs exposure. To date a handful of studies have examined the impact of MPs/NPs on the heart in vertebrates, but most oral exposure studies have used male animals. These studies have demonstrated that MPs in the heart can contribute to elevated inflammation and oxidative stress, apoptosis, and increased cardiac fibrosis, all resulting in cardiac dysfunction (Li et al., 2020; Wei et al., 2021). Since *Drosophila* hearts have homologous genetic and functional changes in development and aging, with the vertebrate heart, it has made them an excellent model for studying molecular mechanisms behind changes in the heart (Wolf et al., 2006; Ocorr et al., 2007; Bryantsev and Cripps, 2009; Blice-Baum et al., 2019) therefore they could prove to be invaluable in the evaluation of MPs/NPs and their role in heart health.

## 2 Methods

### 2.1 Plastic feeding

The *Drosophila* wild-type fly line (W^1118^) was obtained from Bloomington Stock Center, Indiana, USA (BL83009). In this study, flies were reared at 25°C in 60% humidity with a 12 h light/dark cycle. Flies were raised on a standard cornmeal diet.

Polystyrene (PS) NPs and MPs were obtained from Phosphorex Inc. (Hopkinton, MA, catalog numbers 102 and 112, respectively). NPs were 0.041 ± 0.007 μm in diameter, while MPs were 1.051 ± 0.199 μm in diameter (sample mean ± sample standard deviation), as specified by the manufacturer. The density of the polymer is 1.06 g/cm^3^. *Drosophila* larvae were exposed to 0.0783 g of plastic/kg of larvae/d, which is 7.8 times higher than the estimated maximum of what a 70 kg person would consume per day (0.01 g per kg body weight) (Senathirajah et al., 2021). This dose was selected as a worst-case scenario in terms of human dietary exposure. The final estimated number of particles that larvae were exposed to was 1.4 × 10^11^ particles/d/kg of larvae for MPs and 1.2 × 10^18^ particles/d/kg of larvae for NPs. Particles were received as 5 mL volume suspended in ultra-pure DI water. 0.1% of polysorbate surfactant Tween 20 was added to suspension (to prevent clumping) before mixing with food. MPs and NPs were mixed in solid food to demonstrate the effects of developmental oral exposure. The final concentration of Tween 20 in the food was 0.000002%. Tween 20 was added to the control group (no plastic) as well. Flies were reared on plastic containing diet, or control, from the beginning of development through pupation. Newly eclosed adults were immediately removed for analysis. Sorting of flies was done with CO_2_ on a FlyPad, while FlyNap was used to anesthetize flies for heart analysis.

### 2.2 Heart function

To determine any potential effects of plastic exposure on heart function, Semi-Automatic Optical Heart Analysis (SOHA) was used. Flies were reared on previously described treatments (control, 1 μm MPs, and 0.05 µm NPs) and their heart function analyzed. Approximately, 15 flies per sex per treatment were collected 5 days post eclosion from control, NPs, and MPs treatments. After being anesthetized, semi-intact dissections were done as previously established by Vogler and Ocorr (2009). Using NIS-Elements software, 30-s video recordings of the still beating hearts were recorded using an Andor Zyla 5.5 sCMOS high speed camera at 200–210 frames per second. The videos were analyzed, via m-mode generation, with Semiautomatic Optical Heartbeat Analysis (SOHA) software (Ocorr et al., 2009). The raw SOHA output was then analyzed in Excel. Heart function followed Gaussian distribution and was analyzed via One-way ANOVAs and Students T-tests in Excel.

## 3 Results

**FIGURE 1:**
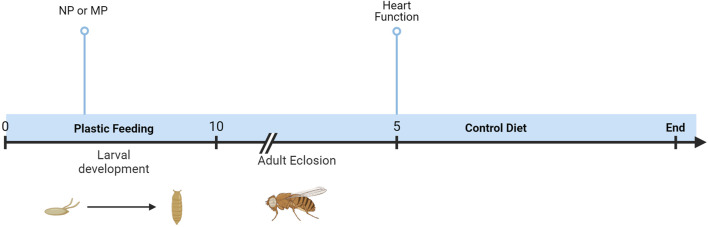
Timeline of experiments. Oral exposure to NPs or MPs was achieved via developmental feeding. Flies were removed from the diet upon eclosion, and heart function was assessed after 5 days.

To explore the impact of plastic on the heart, semi-intact dissections were completed, and function analyzed with SOHA. Developmental dietary exposure to NPs or MPs (Figure 1) results in several sexually dimorphic functional changes to the heart.

**FIGURE 2:**
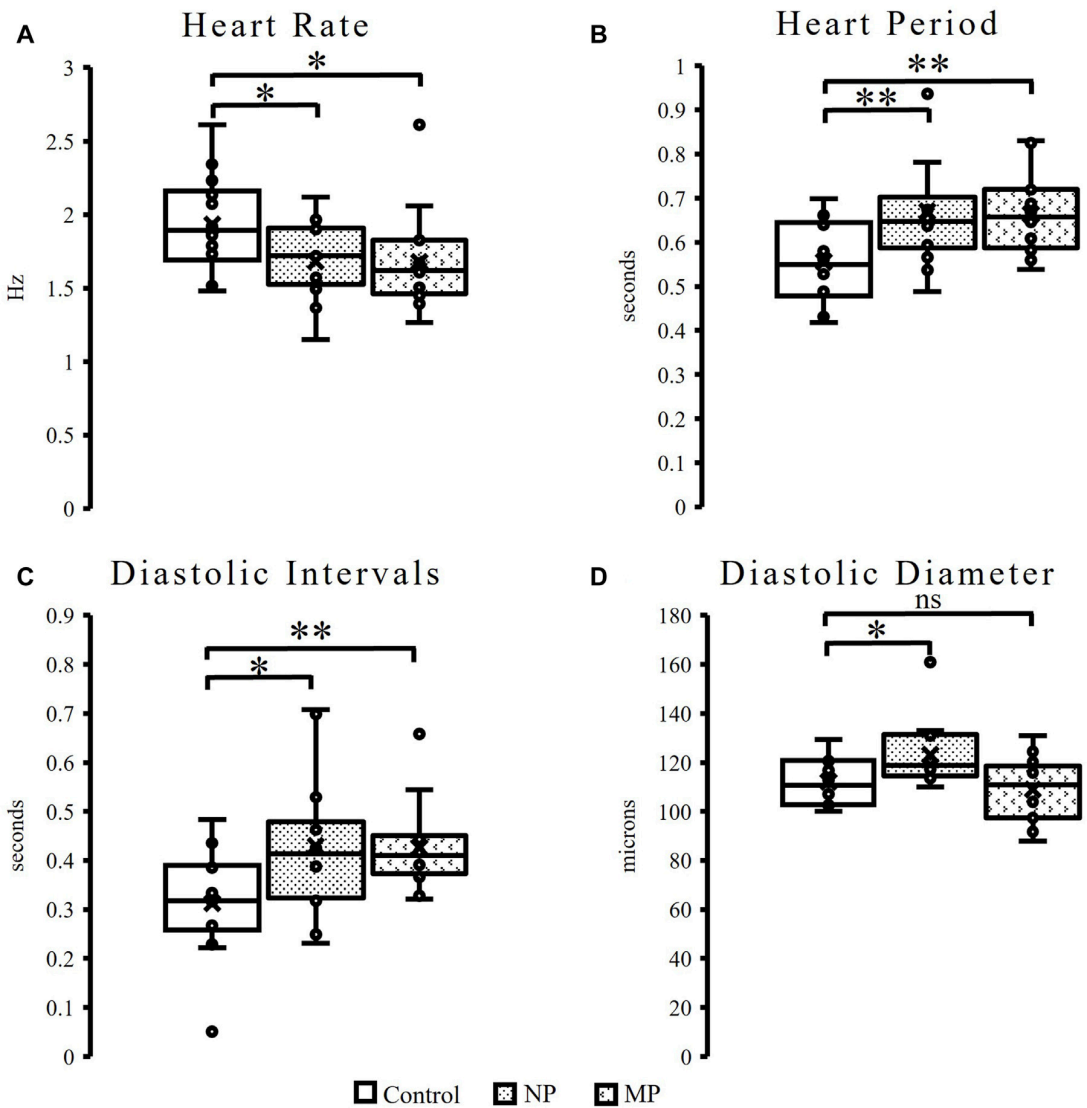
Heart function in female flies. Female flies experience a significant, 13%, decrease in heart rate, (NPs *p* = 0.03, MPs *p* = 0.05) following plastic ingestion **(A)**. Female flies exposed to NPs and MPs also experience a significant increase in heart period (NPs *p* = 0.01, MPs *p* = 0.004) **(B)** and diastolic intervals (NPs *p* = 0.02, MPs *p* = 0.005) **(C)**. Following ingestion of NPs a significant increase in diastolic diameter (*p* = 0.02) is observed **(D)**. p ≤ 0.05*; *p* ≤ 0.01**; *p* ≤ 0.001***.

Female flies with exposure to both plastic sizes experience about 13% decrease in heart rate (Figure 2A, NP *p* = 0.03, MP *p* = 0.05) with the corresponding increase in heart period (Figure 2B, NP *p* = 0.01, MP = 0.004). While no change is seen in male flies to heart rate or heart period (Supplementary Figures S1A, S1B), it should be noted that there is much greater variability seen in the male flies fed MPs compared to control and NPs. Female flies also demonstrate significant increases to diastolic intervals (Figure 2C, NPs *p* = 0.02, MPs *p* = 0.005) and in the diastolic diameter in flies exposed to NP (Figure 2D, *p* = 0.02).

**FIGURE 3:**
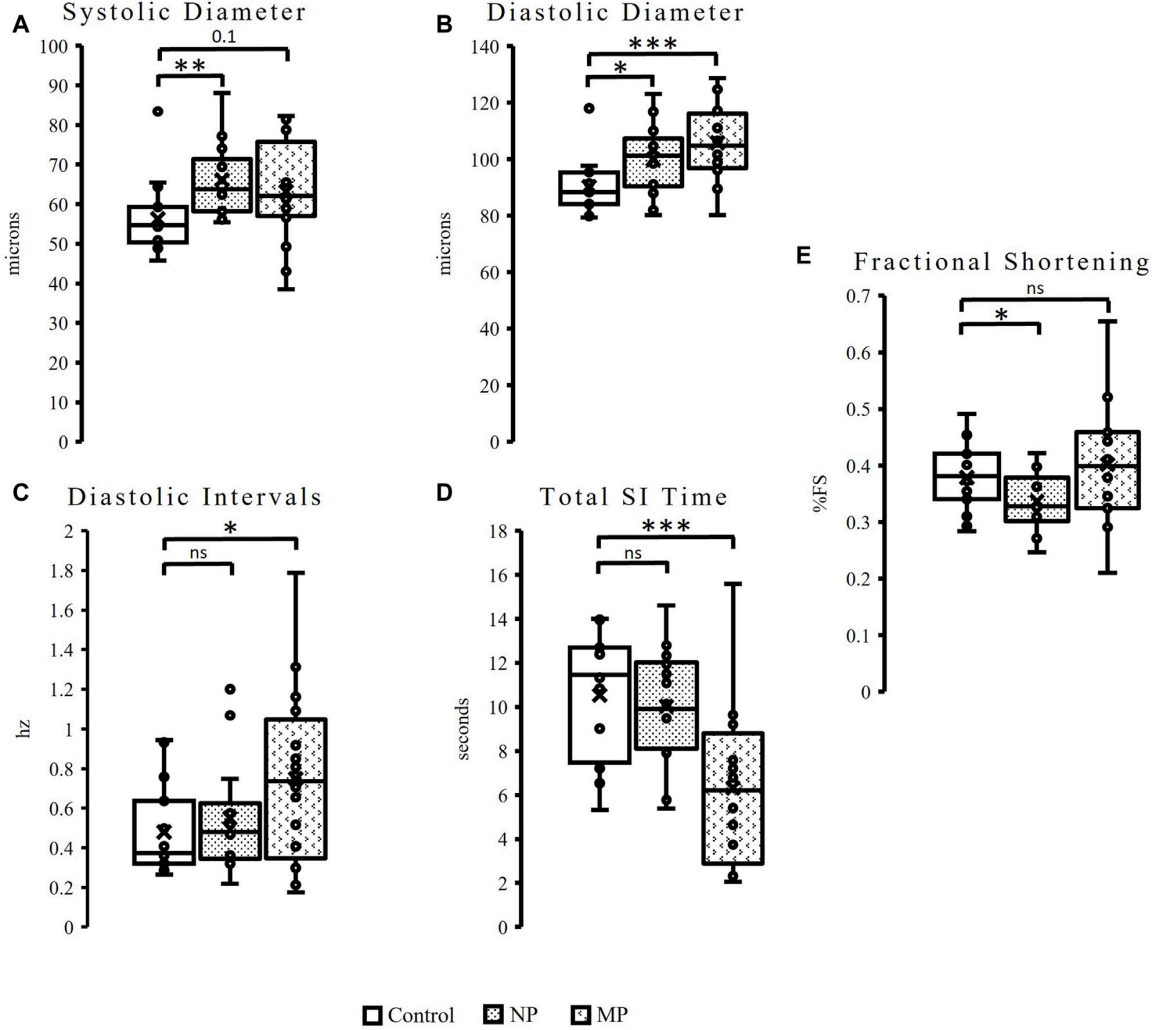
Oral exposure of both plastic types to male flies results in a significantly increased heart size, as seen in the significant, 17%, increase to systolic diameter (*p* = 0.008) following NPs feeding. There is also a slight increase (12%) to systolic diameter (*p* = 0.1) following MPs exposure **(A)**. Additionally significant changes are seen to diastolic diameter, a 10% increase following exposure to NPs (*p* = 0.03), and 17% increase after MPs (*p* = 0.0008) **(B)**. Male flies show a 56% increase Diastolic Intervals (*p* = 0.04) after MPs exposure **(C)**, as well as a 40% reduction in Total Systolic Interval (SI) Time (*p* = 0.001, **(D)**. Fractional Shortening is decreased by 11% following NP (*p* = 0.05) exposure **(E)**. p ≤ 0.05*; *p* ≤ 0.01**; *p* ≤ 0.001***.

Similar to female flies, male flies exposed to dietary plastic exposure also demonstrate changes to heart size (diameter) and diastolic intervals. However, in males, the significant change to heart size is seen in flies fed both plastic sizes and the changes are observed in both diastolic and systolic diameters. Male flies fed NPs show a 17% increase in systolic diameter (*p* = 0.008), while flies fed MPs have a trending (*p* = 0.1), 12%, increase in systolic diameter (Figure 3A). Significant changes are seen in diastolic diameter following both plastic feedings. MPs exposure results in a significant 17% increase in diastolic diameter (*p* = 0.0008) and NPs, a 10% increase in diastolic diameter (Figure 3B, *p* = 0.03). Like females, males also show changes to diastolic intervals (DI). Male hearts exposed to MPs reveal a 56% increase in DI (Figure 3C, *p* = 0.04).

Unlike females, male flies also see changes to Systolic Interval (SI) Time and fractional shortening. Total SI time is reduced by 40% in flies exposed to MPs (Figure 3D, *p* = 0.001) while female flies see no change (Supplementary Figure S2A). Finally, males exposed to NPs experience an 11% reduction in fractional shortening (Figure 3E, *p* = 0.05). This phenomenon is unique to males, as females see no change to fractional shortening following dietary exposure to either plastic size (Supplementary Figure S2B).

## 4 Discussion

This is the first study to evaluate the effects of MPs and NPs on heart function in *Drosophila*. Several studies in mammalian (Geiser et al., 2005; Li et al., 2020; Lim et al., 2021) and fish (Chen et al., 2017; Pitt et al., 2018; Wang et al., 2019) models have observed changes to heart function in response to plastic consumption, however the molecular mechanism behind these changes has yet to be revealed. The results from this study are consistent with findings from other models that demonstrate a variety of negative effects on the heart, including increased heart size (Lim et al., 2021) and decreased heart rate (Pitt et al., 2018; Wang et al., 2019; Li et al., 2020). The female heart function results in this study are consistent with the decrease in heart rate following NPs accumulation in the pericardium of fish (Lett et al., 2021). Male and female flies both showed an increase in heart size, which is consistent with inhalation of NPs (100 nm) in Sprague Dawley rats (Lett et al., 2021). Diastolic intervals are known to have an inverse relationship with heart rate, as heart rate increases diastolic intervals shorten until they disappear completely (Chung et al., 2004), which is a common symptom of diastolic dysfunction. The opposite is observed in female flies in the study. The slowing heart rate and increasing diastolic intervals could suggest hypertrophic cardiomyopathy; however, additional studies would need to be done to confirm. While both NPs and MPs consumption increases diastolic intervals in females, only MPs result in that phenotype in males. Males, interestingly, also show a considerable decrease in total SI time, which is suggestive of systolic dysfunction. Interestingly NPs exposure resulted in a significant drop in fractional shortening, demonstrating a decreased ability for the heart to pump normally.

The study conducted by Alaraby et al. (2022a, 2022b), demonstrated that NPs can translocate into the hemolymph in *Drosophila*, which would allow them to interact with organs, including the heart, directly. So, it is also plausible that the dysfunction seen in the heart could result from changes during development (egg or pupae). An initial hypothesis for this study was that the changes observed may be due to the presence of MPs and NPs in the heart causing a physical barrier to normal development (Jovanović, 2017). Since we see different changes, particularly to heart size (in both males and females), this leads us to believe the changes are a result of molecular interactions between the plastics and the heart itself. The true mechanism behind these observed changes is unknown, and so further research is needed to identify if exposure to MPs and NPs interact with any mammalian conserved genes which may lead to cardiac dysfunction (Piazza and Wessells, 2011). Additionally, it has become apparent that MPs/NPs can have significant impacts on terrestrial insects (Li et al., 2024) and the *Drosophila* model could provide important insights into the effects on insect health. This study was focused on polystyrene beads of two different sizes, but given our observed changes in the heart, additional study which emulates the complexity of plastic shapes that are ingested would be interesting for future research. Additionally, future studies will be needed to determine the molecular changes that could be leading to the observed functional defects.

The results of this study demonstrate specific male and female phenotypes, indicating a sexually dimorphic response, to plastic exposure (summarized in Table 1). Recent research suggests that NPs/MPs have varying effects on males and females, particularly when it comes to fertility (Hong et al., 2023). It is well known that in mammals, the hearts of males and females differ in aging (Strait and Lakatta, 2012) and disease presentation/progression. In *Drosophila* females are significantly larger than males (Viswanathan et al., 2014; Mathews et al., 2017), this difference in size may impact the way in which the particles circulate. Additionally, the two sexes have differences in physiology and development, including growth, cell signaling pathways, metabolism, and organ homeostasis (Millington & Rideout, 2018). Interestingly, we found that MPs were not affecting the diameter of the hearts in female flies; since we see the changes with males (the smaller animal) we can hypothesize that there is an important molecular mechanism that regulates this phenomenon. Taken together the changes, following NPs/MPs exposure, seen in female flies are suggestive of diastolic dysfunction. While the male results are more indicative of systolic dysfunction, as evidenced by the changes to diastolic intervals, systolic interval time and reduced fractional shortening, particularly for NPs exposed flies. Male results also suggest dilated cardiomyopathy as seen by the significant increases seen in heart diameter.

**TABLE 1:** Summary of changes in the hearts of flies exposed to NP/MPs. Sexually dimorphic changes to heart function are seen among age matched male and female flies. Females exhibit more functional changes while males demonstrate functional and morphological changes to the heart following oral exposure to NP/MP. Arrows indicate direction of change, *p* ≤ 0.05*; *p* ≤ 0.01**; *p* ≤ 0.001***.

Female		Male	
Nanoplastic	Microplastic	Nanoplastic	Microplastic
↓ Heart rate*	↓ Heart rate*	Heart rate	Heart rate
↑ Heart period**	↑ Heart period**	Heart period	Heart period
↑ Diastolic Intervals*	↑ Diastolic Intervals*	↑ Diastolic Intervals*	Diastolic Intervals
Total SI Time	Total SI Time	↓ Total SI Time***	Total Si Time
Fractional Shortening	Fractional Shortening	Fractional Shortening	↓ Fractional Shortening*
↑ Diastolic Diameter*	Diastolic Diameter	↑ Diastolic Diameter*	↑ Diastolic Diameter*
Systolic Diameter	Systolic Diameter	↑ Systolic Diameter*	↑ Systolic Diameter ^(0.1)^

In conclusion developmental oral exposure to NPs and MPs results in sexually dimorphic functional changes to the *Drosophila* heart. Doing this work in a highly tractable genetic model, often used to model human disease, where similar changes to what has previously been demonstrated in vertebrate models, has demonstrated the significance of the *Drosophila* model system in plastic toxicology research. The data from this study can inform the field on potential changes to other terrestrial organisms and opens the door to future studies investigating the molecular mechanism behind these changes and underscores the importance of research in plastic on both sexes.

### 4.1 Permission to reuse and copyright

Permission must be obtained for use of copyrighted material from other sources (including the web). Please note that it is compulsory to follow figure instructions.

## Data Availability

The raw data supporting the conclusions of this article will be made available by the authors, without undue reservation.
