# Supplementary figures and images for: The heart of plastic: utilizing the Drosophila model to investigate the effects of micro/nanoplastics on heart function
Source: Front Toxicol. 2024 Aug 16;6:1438061. doi: 10.3389/ftox.2024.1438061 (PMC11362071; doi:10.3389/ftox.2024.1438061)

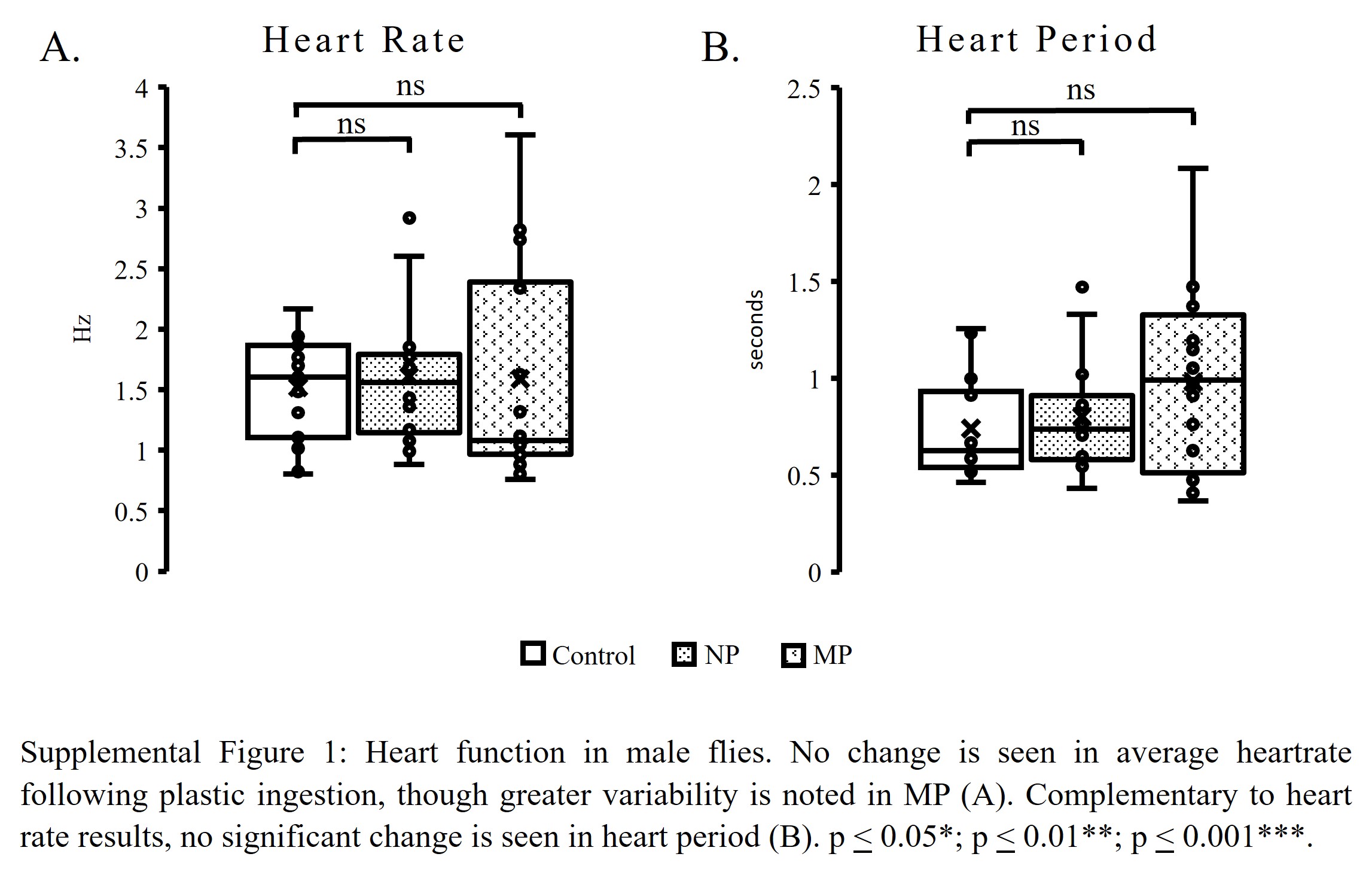

Supplement: Supplementary file 1 [file Image1.jpeg]

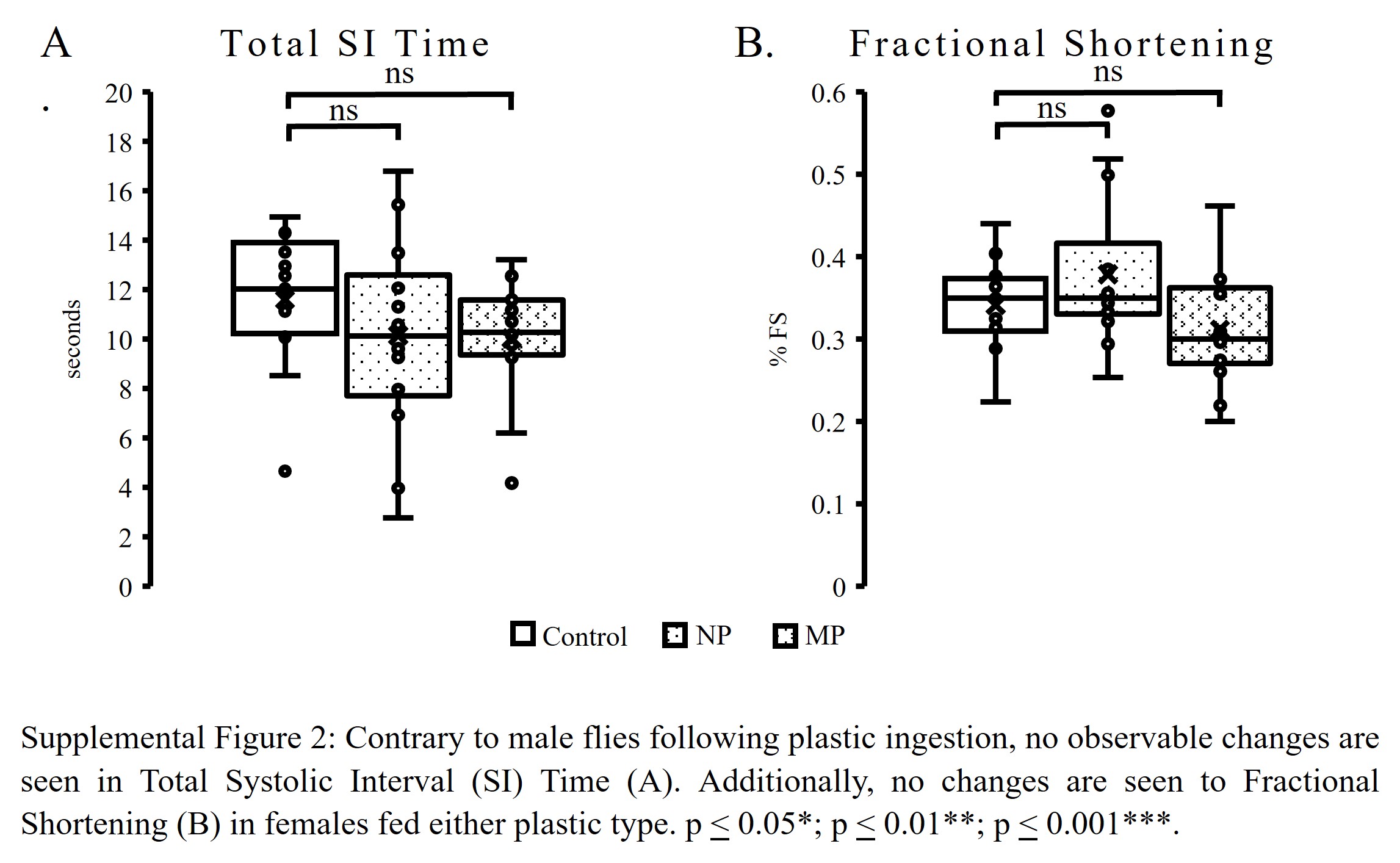

Supplement: Supplementary file 2 [file Image2.jpeg]
